# Dual RNA Sequencing Reveals the Expression of Unique Transcriptomic Signatures in Lipopolysaccharide-Induced BV-2 Microglial Cells

**DOI:** 10.1371/journal.pone.0121117

**Published:** 2015-03-26

**Authors:** Amitabh Das, Jin Choul Chai, Sun Hwa Kim, Kyoung Sun Park, Young Seek Lee, Kyoung Hwa Jung, Young Gyu Chai

**Affiliations:** 1 Department of Bionanotechnology, Hanyang University, Seoul, Republic of Korea; 2 Department of Molecular & Life Sciences, Hanyang University, Ansan, Republic of Korea; 3 Institute of Natural Science & Technology, Hanyang University, Ansan, Republic of Korea; Massachusetts General Hospital and Harvard Medical School, UNITED STATES

## Abstract

Microglial cells become rapidly activated through interactions with pathogens, and the persistent activation of these cells is associated with various neurodegenerative diseases. Previous studies have investigated the transcriptomic signatures in microglia or macrophages using microarray technologies. However, this method has numerous restrictions, such as spatial biases, uneven probe properties, low sensitivity, and dependency on the probes spotted. To overcome this limitation and identify novel transcribed genes in response to LPS, we used RNA Sequencing (RNA-Seq) to determine the novel transcriptomic signatures in BV-2 microglial cells. Sequencing assessment and quality evaluation showed that approximately 263 and 319 genes (≥ 1.5 log_2_-fold), such as cytokines and chemokines, were strongly induced after 2 and 4 h, respectively, and the induction of several genes with unknown immunological functions was also observed. Importantly, we observed that previously unidentified transcription factors (TFs) (irf1, irf7, and irf9), histone demethylases (kdm4a) and DNA methyltransferases (dnmt3l) were significantly and selectively expressed in BV-2 microglial cells. The gene expression levels, transcription start sites (TSS), isoforms, and differential promoter usage revealed a complex pattern of transcriptional and post-transcriptional gene regulation upon infection with LPS. In addition, gene ontology, molecular networks and pathway analyses identified the top significantly regulated functional classification, canonical pathways and network functions at each activation status. Moreover, we further analyzed differentially expressed genes to identify transcription factor (TF) motifs (−950 to +50 bp of the 5’ upstream promoters) and epigenetic mechanisms. Furthermore, we confirmed that the expressions of key inflammatory genes as well as pro-inflammatory mediators in the supernatants were significantly induced in LPS treated primary microglial cells. This transcriptomic analysis is the first to show a comparison of the family-wide differential expression of most known immune genes and also reveal transcription evidence of multiple gene families in BV-2 microglial cells. Collectively, these findings reveal unique transcriptomic signatures in BV-2 microglial cells required for homeostasis and effective immune responses.

## Introduction

Neuroinflammation is a key mechanism against infectious agents and neuronal injuries in the central nervous system (CNS). However, uncontrolled neuroinflammatory reactions lead to the neuronal damage observed in many neurodegenerative disorders, such as Alzheimer’s, Parkinson’s, Huntington’s, and Multiple sclerosis diseases [1]. Microglial cells form approximately 10–20% of cells in the CNS, and these specialized macrophage-like immune cells are involved in the initiation of innate immune responses [2]. Microglial cells are highly mobile and rapidly activated through various neuronal injuries, stresses, and infections. The activated microglia also release various inflammatory mediators, including tumor necrosis factor-alpha (tnf-α), interleukin (il)-1β, il-6, nitric oxide (NO), reactive oxygen species (ROS), and prostaglandin E2 (pge2), which could be neurotoxic [3]. Although microglial activation is essential for host defense in the brain, the abnormal activation of microglia can lead to devastating outcomes, such as neuroinflammation, a major cause of neurodegenerative diseases [4]. Therefore, understanding the regulation of microglial activation using genome-wide approaches is required to obtain greater insight into the repertoire of LPS-stimulated gene expression profiling in BV-2 microglial cells involved in neuroinflammatory disorders.

Microglial cells are activated in response to environmental stress, lipopolysaccharide (LPS), interferon (IFN)-γ and β-amyloid [4]. LPS is a heat-stable, amphiphilic molecule comprising three regions, namely lipid A, the polysaccharide core, and an O-specific side chain, and this molecule is ubiquitously observed in most environments, such as cigarettes, contaminated foods and medicine, and non-sterile water [5–8]. Many serious inflammatory diseases, including sepsis, neurodegenerative diseases, pneumonia, and so on, are induced through LPS [9, 10]. LPS, the main component of endotoxins, has been isolated from Gram-negative bacteria and employed to induce microglial activation and initiate several major cellular responses that play important roles in the pathogenesis of inflammation [11]. Thus, the LPS-mediated stimulation of microglia is a useful model to study the mechanisms underlying neuronal damage mediated through pro-inflammatory and neurotoxic factors, such as NO, pge2, ROS, il-1β, il-6 and tnf-α, released from activated microglia [12, 13].

To date, several genome-scale studies of LPS-induced BV-2 microglial cells have been conducted to determine comprehensive signatures using the microarray method [14–16]. However, this method has numerous restrictions, such as spatial biases, uneven probe properties, low sensitivity, and dependency on the probes spotted [17–19]. Next generation sequencing (NGS)-based technologies, such as RNA-Seq, are increasingly used to study gene expression, as these methods provide unbiased profiles, identify novel transcribed regions compared with microarrays, and can be extremely accurate when a sufficient coverage is obtained. Furthermore, these technologies facilitate the differentiation between the expression of alternative mature mRNAs from the same precursor and the identification of the differential expression of mRNA isoforms [20–22]. Validation techniques, such as qRT-PCR [23], have corroborated the accuracy of RNA-Seq; however, a limited number of studies have applied these approaches for the effects of endotoxin infection on changes in global gene expression in macrophages using RNA-Seq analysis [24, 25]. Thus, the objective of the present study was to understand host responses to LPS infection in cultured microglial cells using RNA-Seq analysis.

In the present study, we implemented RNA-Seq approaches to characterize global transcriptional responses at 2 and 4 h in BV-2, a microglia cell line, infected with LPS. We demonstrated that LPS infection strongly induced the expression of the genes involved in innate immune responses. The obtained data confirmed that many signaling pathways are potentially involved in the host immune response to LPS infection. We performed TF motif analysis of the promoter regions of genes exhibiting differential gene expression under the tested conditions and identified binding sequences for known TFs. Furthermore, we also highlighted many differences in splicing isoforms, TSSs and differential promoter usage in LPS-stimulated BV-2 microglial cells. The pattern of innate immune responses in LPS infection might enhance the current understanding of the pathogenesis of endotoxins and improve therapeutic methods. In this context, the present study provides a reliable and representative model for the accurate characterization of the development and regulation of microglia functions during an inflammatory stimulus.

## Materials and Methods

### Cell culture and stimulation

Mouse microglia BV-2 cells were grown in high glucose Dulbecco’s Modified Eagle’s Medium (DMEM) supplemented with 10% Fetal Bovine Serum (FBS) (catalog # 26140), 100 IU/ml penicillin, and 10 μg/ml streptomycin (catalog # 15140; Invitrogen, USA). The murine microglial cell line BV-2 was a kind gift from Dr. Hee-Sun Kim (Ewha Womans University, Department of Molecular Medicine, Tissue Injury Defense Research Center, Seoul, South Korea) [26]. The mouse macrophage cell line, RAW 264.7, was obtained from the American Type Culture Collection (Manassas, VA), and the cells were grown in RPMI medium supplemented with FBS, 100 IU/ml penicillin, and 10 μg/ml of streptomycin (Invitrogen, USA). The cells were maintained in a humidified incubator with 95% air and a 5% CO_2_ atmosphere at 37°C. BV-2 and RAW 264.7 cells were incubated with LPS (10 ng/mL, Sigma Aldrich) for the specified times under normal culture conditions. The medium, containing the appropriate agents, was replaced every other day. β amyloid peptides, the 42 aa (Aβ_42_) (purity >95%) was purchased from Alpha Diagnostic International (San Antonio, Texas, USA). Unless otherwise indicated, Aβ_42_ was freshly dissolved before use in the experiments. In this study, we used *nanomolar concentrations* of Aβ_42_ (225 ng/mL) [27]. Primary microglial cells were isolated from 3-day-old ICR mice as previously described [28]. All experimental protocols were conducted in accordance with Institutional Animal Care and Use Committee (IACUC) guidelines and were approved by the IACUC committee at Hanyang University (HY-IACUC-2014-0164A). Briefly, whole brains of neonatal mice were taken; blood vessel and meninges were carefully removed. Then, the whole brains of 12 mice were pooled together, finely minced, and digested with Neural Tissue Dissociation Kit-Postnatal Neurons (Miltenyi Biotec-130-094-802). Next, digested cells pass through 70 um nylon cell strainer (BD Biosciene) and were seeded in poly-L-lysine-coated T-75 flask in DMEM/nutrient mixture F-12 (DMEM/F12, 1:1) containing 20% FBS (catalog # 26140), 100 IU/ml penicillin and 10 μg/ml streptomycin (catalog # 15140) from Invitrogen (CA, USA). The cells were maintained in a humidified incubator with a 95% air/5% CO_2_ atmosphere at 37°C. The medium was changed every 2–3 days. After two weeks in culture, mixed glial cell cultures are shaken at 150 rpm at 37°C for 45 min, and the glial cell suspension was collected from each flask and seeded on poly-L-lysine coated cell culture plate. Microglial cells were sub plated and used for further experiments. More than 96% of cells obtained were microglia as quantified by CD11b (rat monoclonal immunoglobulin G2b (IgG2b), clone M1/70.15.11.5, Miltenyi Biotec Inc., Auburn, CA, USA) FACS analysis (S1 Fig.).

### Total RNA extraction

Total RNA (~8 μg) was extracted using TRIzol (Life Technologies) according to the manufacturer’s instructions. Briefly, two hundred microliters of chloroform was added, and the tubes with the lysis mixture were gently inverted for 5 minutes (min). The mixture was centrifuged at 12,000 x *g* for 15 min at 4°C, and the clear upper solution was placed into a new tube, to which 500 μl of isopropanol was added. The tubes were inverted before incubation on ice for 1 h. The lysis mixture was centrifuged at 12,000 x *g* for 10 min at 4°C, and the isopropanol was decanted. Ice-cold 70% ethanol was added to the RNA pellet for gentle washing. After centrifuging as indicated above for 10 min, the ethanol was removed. The RNA pellets were dried at room temperature for 5–10 minutes before reconstitution in 20 μl RNase-free water, and the RNA was treated with RNase-free DNase I (Promega, Wisconsin, USA). The RNA quality was assessed using an Agilent 2100 Bioanalyzer with an RNA 6000 Nano Chip (Agilent Technologies, Waldbronn, Germany), and the quantity was determined using a spectrophotometer (NanoDrop Technologies, Wilmington, DE, USA).

### Quantitative real-time RT-PCR

The reverse transcription of the RNA samples was performed as previously described [29] using 2 μg of total RNA, 1 μl of random hexamers (per reaction) and the PrimeScript 1st-strand cDNA Synthesis Kit (Takara, Japan). The random hexamers and RNA templates were mixed and denatured at 65°C for 5 min., followed by cooling for 2 min on ice. PrimeScript buffer (5x), RTase and RNAse inhibitor were added to the cooled template mixture and incubated for 1 h at 50°C before enzyme inactivation at 70°C for 15 min. Quantitative real-time RT-PCR (qRT-PCR) was performed using SYBR Green PCR Master Mix (Takara Bio Inc., Shiga, Japan) and a 7500 fast real-time PCR system (Applied Biosystems, Foster City, USA). Glyceraldehyde-3-phosphate dehydrogenase (GAPDH) was used as an internal control. Complementary DNA samples were diluted 1.5-fold, and qRT-PCT was performed using an AB-7500 Real-time thermal cycler (Applied Biosystems, Foster City, USA) with SYBR Premix Ex-Taq II (Takara Bio, Shiga, Japan) according to the manufacturer’s instructions. The reactions were performed in a total volume of 20 μl containing 0.4 mM of each primer (Table 1). Each PCR run included a no-template control including water instead of cDNA and a reverse transcriptase-negative control for each gene. Triplicate measurements were performed for all reactions. Different samples were evaluated using 96-well plates for gene expression experiments, and all samples were analyzed on a single plate for endogenous control determination. The results were analyzed using the critical threshold (ΔC_T_) and comparative critical threshold (ΔΔC_T_) methods in the AB-7500 software program with the Norm finder and geNorm-plus algorithms. The primers were designed using Primer Express (Applied Biosystems, Foster City, USA).

**Table 1:** List of primers used in q-RT-PCR studies.

Gene designation	Forward (5' -> 3')	Reverse (5' -> 3')
***Tnf-α***	CAG GCG GTG CCT ATG TCT C	CGA TCA CCC CGA AGT TCA GTA G
***Il1b***	GAA ATG CCA CCT TTT GAC AGT G	CTG GAT GCT CTC ATC AGG ACA
***Cxcl10***	TGC TGG GTC TGA GTG GGA CT	CCC TAT GGC CCT CAT TCT CAC
***Relb***	CCG TAC CTG GTC ATC ACA GAG	CAG TCT CGA AGC TCG ATG GC
***iNOS***	ACA TCG ACC CGT CCA CAG TAT	CAG AGG GGT AGG CTT GTC TC
***p65***	AGG CTT CTG GGC CTT ATG TG	TGC TTC TCT CGC CAG GAA TAC
***Irf-9***	CCTCAGGCAAAGTACGCTG	GGGGTGTCCTATGTCCCCA
***Irak-3***	GTTCTACTCCTGTTCCGTCACC	GTCCCGTTGCTCATATAGGGATA
***Ccl-12***	ATTTCCACACTTCTATGCCTCCT	ATCCAGTATGGTCCTGAAGATCA
***Irf-1***	ATG CCA ATC ACT CGA ATG CG	TTG TAT CGG CCT GTG TGA ATG
***Irf-7***	GCGTACCCTGGAAGCATTTC	GCACAGCGGAAGTTGGTCT
***Ccl-7***	CCACATGCTGCTATGTCAAGA	ACACCGACTACTGGTGATCCT
***Ccl-2***	TAA AAA CCT GGA TCG GAA CCA AA	GCA TTA GCT TCA GAT TTA CGG GT
***Ptgs-2***	TTCCAATCCATGTCAAAACCGT	AGTCCGGGTACAGTCACACTT
***Il1a***	TCTATGATGCAAGCTATGGCTCA	CGGCTCTCCTTGAAGGTGA
***Irg-1***	GGCACAGAAGTGTTCCATAAAGT	GAGGCAGGGCTTCCGATAG
***Ccl-4***	TTCCTGCTGTTTCTCTTACACCT	CTGTCTGCCTCTTTTGGTCAG
***c-REL***	AGA GGG GAA TGC GGT TTA GAT	CTA CCT GCT GAT CGC CCT TC
***Gapdh***	TGCGACTTCAACAGCAACTC	CTTGCTCAGTGTCCTTGCTG

### cDNA library preparation for RNA-Seq

Total RNA was extracted from each group of BV-2 cells (i.e., control 2 h, control 4 h, LPS 2 h, LPS 4 h) using TRIzol (Life Technologies) according to the manufacturer’s instructions. For RNA-Seq, RNA libraries were created from each group of BV-2 cells using the NEBNext Ultra Directional RNA Library preparation kit from Illumina. The first step in the workflow involved the removal of ribosomal RNA using the RNAMius Transcriptome Isolation kit (Life Technologies). Following purification, total RNA was fragmented into small pieces using divalent cations at elevated temperatures. The cleaved RNA fragments were subjected to first-strand cDNA synthesis using reverse transcriptase and random primers, followed by second-strand cDNA synthesis using DNA polymerase I and RNase H. The cDNA fragments were subsequently processed using an end-repair reaction after the addition of a single ‘A’ base, followed by adapter ligation. The products of these reactions were purified and enriched through PCR to generate the final cDNA library. The cDNA fragments were sequenced using the Illumina HiSeq2500 (101 cycles PE lane) (National Instrumentation Center for Environmental Management in Seoul National University). Biological duplicate RNA sequencing was performed on eight independent RNA samples from the treated BV-2 cells: control 2 h (2 samples), control 4 h (2 samples), LPS 2 h (2 samples) and LPS 4 h (2 samples).

### Differential gene expression analysis

Raw sequence files were subjected to quality control analysis using FastQC (http://www.bioinformatics.babraham.ac.uk/projects/fastqc/). To avoid low-quality data, we clipped the adapters and trimmed the reads using the FASTX-Toolkit (http://hannonlab.cshl.edu/fastx_toolkit/). For the analysis of differentially expressed genes, the quality-checked reads for each condition were processed using the TopHat version 2.0.10 package (Bowtie 2 version 2.2.1) as FASTQ files [30]. The reads were mapped to the reference genome [*Mus musculus* UCSC mm10 sequence], and the alignment files were generated as BAM files. These files were used as the input for Cufflinks, a complement method used to generate assembled transcripts for each condition; the abundance was evaluated using read data. To normalize the data, the ‘‘fragments per kilobase per million map reads” (FPKM) were calculated for each gene [31]. These assemblies are used in Cuffquant and Cuffdiff from the Cufflinks 2.2.1 package to calculate the differential expression levels and evaluate the statistical significance of detected alterations [32]. RNA-Seq experiments were normalized and visualized using HOMER (http://homer.salk.edu/homer/) after preparing custom tracks for the UCSC Genome Browser (http://genome.ucsc.edu/). The acquired data were deposited in the Gene Expression Omnibus database under dataset accession no. SRX683618 (SRR1554368, SRR1598823); SRX683736 (SRR1554452); SRX683740 (SRR1554456); SRX683671 (SRR1554369, SRR1598824); SRX683737 (SRR1554453); SRX683741 (SRR1554457).

### Functional annotation

DAVID (Database for Annotation, Visualization and Integrated Discovery) version 6.7 software (http://david.abcc.ncifcrf.gov/home.jsp) was used to determine the most functional annotation of significant genes in the datasets as previously described [33]. **The** DAVID program calculates a modified Fisher’s exact P value to demonstrate gene ontology (GO) or molecular pathway enrichment. Values less than 0.05 were considered strongly enriched in the annotation category.

### Canonical pathway analysis of datasets

IPA (Ingenuity W Systems, http://www.ingenuity.com, Mountain View, CA, USA) was conducted to analyze the most significant canonical pathways in datasets as previously described [29,34,35]. Genes from datasets associated with canonical pathways in the Ingenuity Pathways Knowledge Base (IPAKB) were considered for literary analysis. The significance of the associations between datasets and canonical pathways was measured in the following manner: (1) the ratio of the number of genes from the dataset that mapped to a canonical pathway divided by the total number of genes that mapped to the same canonical pathway; and (2) Fischer’s exact test for a *p* value indicating the probability that the association could be explained by chance. After uploading the datasets, gene identifiers were mapped to corresponding gene objects, and the genes were overlaid onto a global molecular network in IPAKB. Gene networks were algorithmically generated based on connectivity.

### Graphical representation of networks and pathways

For network generation, the molecules from the normalized filtered RNA-Seq dataset were each mapped to corresponding objects in Ingenuity’s Knowledge Base. A fold-change cutoff of up-regulated genes (≥ 1.5 log_2_-fold) was set to identify significantly and differentially regulated genes in BV-2 microglial cells at 2 and 4 h after LPS stimulation. The graphical representation of molecular relationships between genes and gene products was based on genes or gene products, represented as nodes, and the biological relationship between two nodes was represented as an edge (line). All edges were supported by at least one reference from the literature, textbook or canonical information in IPAKB. The node color intensity indicated the degree of up-regulation (red). The nodes were displayed using shapes to represent functional classes of gene products.

### Transcription factor binding motif enrichment analysis

NCBI reference sequence mRNA accession numbers were subjected to transcription factor binding motif analysis using the web-based software Pscan (http://159.149.109.9/pscan/) [36]. The JASPAR [37] database of transcription binding factor sequences was analyzed using enriched groups of −950 base pair (bp) sequences to +50 bp of the 5’ upstream promoters. The range of −950 to +50 was selected from the range options in Pscan to obtain the best cover for a −1000 to +50 bp range.

### Enzyme-linked immunosorbent assay (ELISA)

Primary microglial cells were cultured in the same condition as above. Primary microglial cells were treated with LPS (10 ng/ml), for 2 h and 4 h. After treatment, the concentration of the pro-inflammatory mediators ccl2, ccl7, and cxcl10 were determined in cell culture supernatants using the mouse ELISA kit (Komabiotec, Seoul, Korea) according to the manufacturer’s protocol.

### Statistical analysis

The data were analyzed using Origin Pro 8 (Origin Lab Corporation, Northampton, MA, USA). Each value is expressed as the mean ± standard error of the mean (SEM). The statistical analysis was performed using SPSS 17.0 (SPSS Inc., IL, USA). The data were tested using a one-way ANOVA, followed by Tukey’s HSD post hoc test. ^*^ *P <0*.*05* and ^**^ *P <0*.*001* were considered significant.

## Results

### Effects of LPS on cytokine mRNA induction and the inducer removal response in BV-2 microglial cells

**Fig 1:**
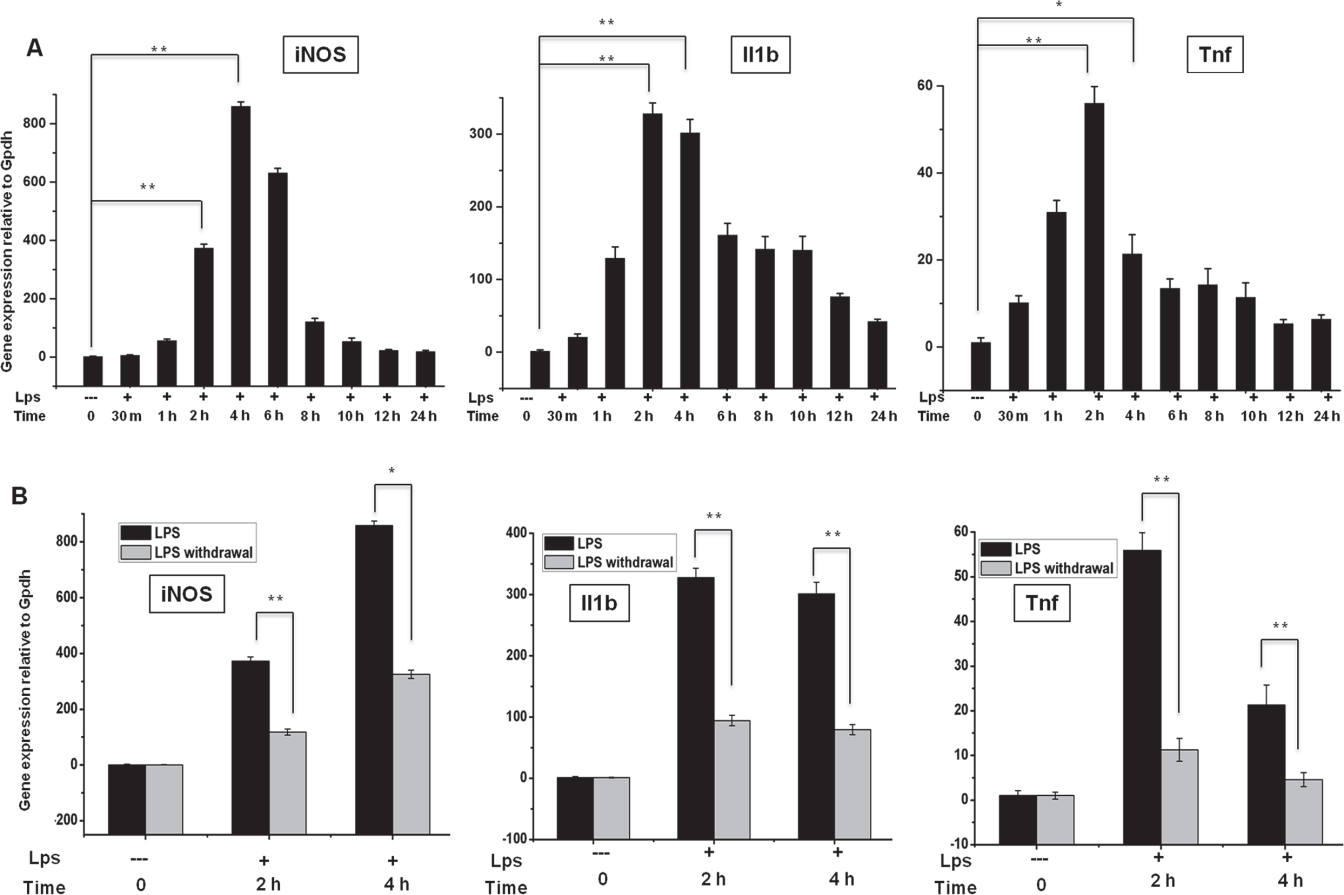
Inflammatory gene expression patterns in response to LPS stimulation and after LPS withdrawal in BV-2 microglial cells. (A, B) Quantitative real-time reverse transcriptase-PCR analysis of inflammatory gene expression in BV-2 microglial cells stimulated with LPS (10 ng/ml) and after LPS was washed away (LPS withdrawal). The expression of inflammatory genes was significantly up-regulated in cells treated with LPS and significantly decreased after the removal of LPS compared with untreated cells (*P<0.05 and **P<0.*001*) at the indicated times. Gene expression was normalized to GAPDH transcript levels. The data represent three independent experiments. The values are shown as the means ± SD of triplicate wells.

In RNA-Seq studies, the preparation of biological samples is a significant initial step. To determine the proper time course responses, we performed an expression analysis in BV-2 microglial cells treated with LPS (10 ng/mL) for 10 min to 24 h and compared the results with the expression in untreated cells under normal culture conditions. We observed the significant, time-dependent up-regulation of inflammatory response-related genes after LPS treatment (Fig. 1A), except for the 12 and 24 h time points. The qRT-PCR analysis revealed that the genes related to inflammatory responses that were up-regulated after LPS treatment in BV-2 microglial cells included iNOS, Il1ß, and tnf-α. We observed that most of the inflammatory response-related genes were up-regulated at the 2 and 4 h time points. Therefore, we selected these time points for transcriptional profiling; these time points were also used in previous studies [14, 38] investigating the general induction patterns of microglial activation through LPS. To determine whether LPS stimulation is essential for maintaining inflammatory gene expression, we further examined the changes in gene expression in response to LPS removal. We removed LPS after 2 and 4 h of treatment and extensively washed the cells, followed by incubation for the specified times under normal culture conditions. The expression of most of the inflammatory genes was suddenly terminated (Fig. 1B), suggesting that LPS stimulation is essential for maintaining inflammatory gene expression in BV-2 microglial cells.

### RNA-seq transcriptional profiling in LPS-stimulated BV-2 microglial cells

**Fig 2:**
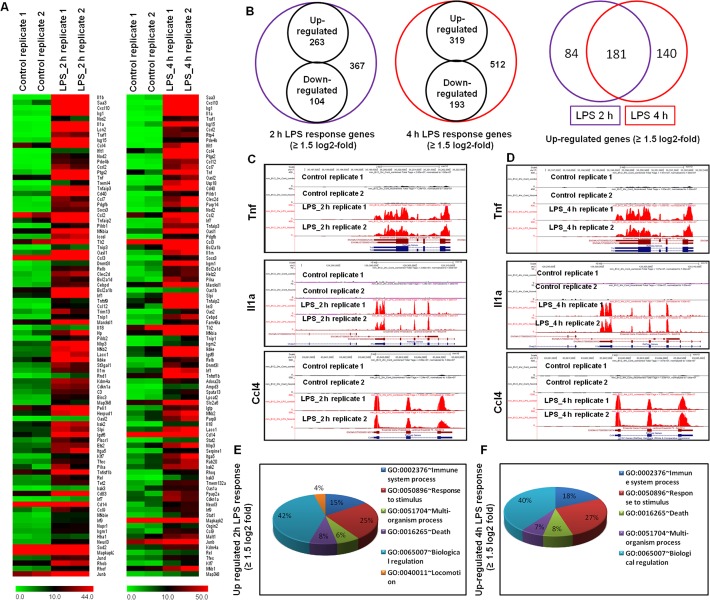
RNA-Seq analysis reveals that LPS-stimulated pro-inflammatory gene expression in BV-2 microglial cells. (A) Heat map representing RNA-Seq gene expression of up-regulated (≥ 1.5 log_2_-fold) inflammatory genes at 2 and 4 h after LPS stimulation in BV-2 microglia cells compared with controls. (B) Venn diagram displaying the number of inducible or repressible (≥ 1.5 log_2_-fold) genes after LPS stimulation in BV-2 microglia cells. (C, D) UCSC Browser images representing normalized RNA-Seq read densities at 2 and 4 h after LPS stimulation in BV-2 microglia cells compared with controls. (E, F) Gene Ontology analysis of functional annotations associated with up-regulated genes at 2 and 4 h after LPS stimulation in BV-2 microglia.

Based on the results shown in Fig. 1A, we treated BV-2 microglial cells with LPS for 2 and 4 h in the cDNA library preparation for RNA-Seq experiments. The RNA-Seq transcriptional analysis was performed using two independent samples (biological replicates) of each treatment. Eight libraries obtained from control 2 h (2 samples), control 4 h (2 samples), LPS 2 h (2 samples) and LPS 4 h (2 samples) treatments were sequenced. The RNA-Seq analysis revealed differentially expressed genes in LPS-stimulated BV-2 cells at both time points: 367 genes for 2 h and 512 genes for 4 h (increased and decreased in expression ≥ 1.5 log_2_-fold, respectively) were differentially regulated. Among them, 263 and 319 genes were up-regulated, whereas 104 and 193 genes were down-regulated at 2 and 4 h, respectively, after LPS treatment (Figs. 2A and 2B and Tables A and B in S1 File). The normalized LPS-stimulated BV-2 microglial cell RNA-Seq data have been deposited in the NCBI Sequence Read Archive [39] and are accessible through accession number SRX683618; SRX683736; SRX683740; SRX683671; SRX683737; SRX683741. The following inflammatory response- and immune response-related genes exhibited the most dramatic levels of induction following LPS challenge: iNOS, interleukin and interleukin-related genes (Il1-β, Il1a, Il18, and Il1rn); Tnf and Tnf-related genes (tnf-α, tnfaip3, tnip3, tnip1, and tnfaip2); a prostaglandin-related gene, ptgs2; NF-κB-related genes (nfκbiz, nfκbia, nfκb2, relb, nfκbie, and nfκb1); interferon-related genes Ifit1, Interferon regulatory factors (irf1, irf7, and irf9); and cytokines or chemokines (cxcl10, ccl4, ccl7, ccl2, ccl3, ccl12, and ccl9) (Figs. 2A, 2C and 2D). We selected these genes based on biological processes and molecular gene ontology functions. As the down-regulated genes were not associated with inflammation, only the up-regulated genes were further studied. We confirmed by gene ontology (GO) analysis (FDR 0.05) using DAVID Bioinformatics Resources that LPS down-regulated transcripts were associated with regulation of biological and cellular processes in BV-2 microglial cells (S2 Fig.). We next performed functional classification analyses of the up-regulated genes (≥ 1.5 log_2_-fold) using DAVID Informatics Resources through classification into GO categories (FDR 0.05) based on biological process (BP) and molecular function (MF) categories and KEGG (Kyoto Encyclopedia of Genes and Genomes) pathways. The genes up-regulated in response to LPS stimulation were involved in several BPs and MFs. We observed that the largest groups of genes were involved in immune system regulation and stimulus responses. Other pathways, such as the regulation of cell death, locomotion, biological processes, etc., were also identified in the analysis of differentially expressed genes at 2 and 4 h after LPS stimulation in BV-2 microglial cells (Figs. 2E and 2F).

### Differential expression of TFs in multiple families of RNA-Seq data in LPS-stimulated BV-2 microglial cells

**Fig 3:**
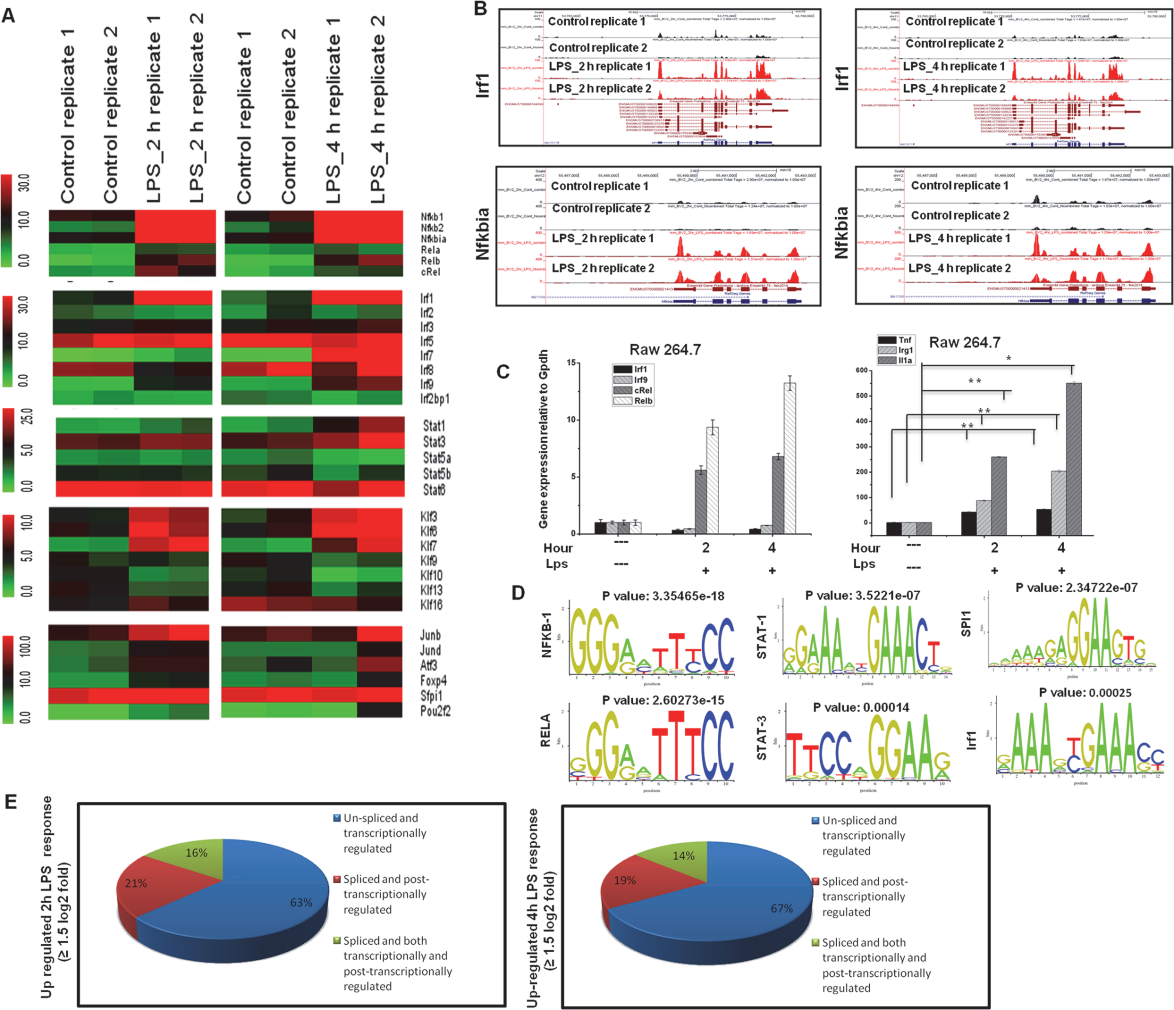
Transcriptomic analysis of selected TF families in BV-2 microglial cells. (A) Heat map represents differential expression of TF families of nfκb, irf, stat, klf, and other genes at 2 and 4 h after LPS stimulation in BV-2 microglial cells. (B) UCSC Browser images representing normalized RNA-Seq read densities for TF expression at 2 and 4 h after LPS stimulation in BV-2 microglia cells compared with controls. (C) Quantitative real-time reverse transcriptase-PCR analysis of TF and inflammatory gene expression in RAW 264.7 mouse macrophage cells stimulated with LPS (10 ng/ml) at the indicated times. (D) Patterns of transcription factor motif enrichments within the promoters of the genes in LPS-stimulated BV-2 microglia cells. (E) Transcriptional and post-transcriptional regulatory effects on overall transcript output in LPS-stimulated BV-2 microglial cells.

Multiple families of TFs were identified among differentially expressed genes (DEGs) that were significantly up-regulated at 2 and 4 h after LPS stimulation in BV-2 microglial cells (Fig. 3A). These TFs, including Irf, Kruppel-like factor (klf), NF-κB and signal transducer and activator of transcription (Stat), are important in neuroinflammatory diseases [40–42]. The RNA-Seq analysis revealed that nfκbia, stat1, klf7 and irf1 were most up regulated in BV-2 microglial cells after LPS stimulation. Interestingly, irf2, irf4, irf6, irf8, stat6, klf1, klf2, klf4, and klf5 were unaffected after LPS treatment, suggesting that LPS-induced gene expression is highly selective in BV-2 microglial cells (Figs. 3A and 3B). To determine whether multiple TF families are uniquely expressed in microglial cells, we compared these highly expressed TFs in macrophage (RAW 264.7) cell lines, and observed that only Irfs (irf1 and irf9) were not expressed in macrophages, suggesting that irf1, irf7, and irf9 might be important regulators in the selective inflammatory gene expression in microglia. However, we could not significantly distinguish other transcription factor groups, such as nf-kb and klf7, and proinflammatory gene expression programs between microglial and macrophages (Fig. 3C). In addition, the RNA-Seq reads also revealed that junb, atf3, foxp4 and specificity protein 1 (spi1) were particularly up regulated in LPS-stimulated BV-2 microglial cells (Fig. 3A). We next conducted a TF motif analysis to assess LPS-induced gene expression in BV-2 microglial cells. We used the Pscan software tool [36] to perform the in silico computational analysis of over represented cis-regulatory elements within the 5’-promoter regions of coordinately regulated genes. Applying this score to the promoters of the genes differentially expressed at 2 or 4 h (≥ 1.5 log_2_-fold) in response to LPS revealed that the DNA sequences not only for NF-kB transcription factors but also for irf1, stat1, stat3 and Spi1 were significantly enriched (Fig. 3D). These findings indicate that multiple families of transcription factors might be involved in the regulation BV-2 microglial cell activation.

### Transcriptional and post-transcriptional regulation in LPS-stimulated BV-2 microglial cells

Differentially expressed isoforms with different TSSs are transcriptionally regulated, while differentially expressed isoforms with the same TSSs are post-transcriptionally regulated [25]. In the present study, the transcripts, isoforms and TSSs of the genes up-regulated (≥ 1.5 log_2_-fold) in LPS-stimulated BV-2 cells after 2 and 4 h were investigated. We defined three groups of genes (Fig. 3E and Tables 2 and 3): A. Genes with one TSS and one isoform, classified as ‘‘un-spliced and transcriptionally regulated” (163 and 216 genes, respectively); B. Genes with one TSS and more than one isoform, classified as ‘‘spliced and post-transcriptionally regulated” (55 and 63 genes, respectively); and C. Genes with more than one TSS and more than one isoform, classified as ‘‘spliced and both transcriptionally and post-transcriptionally regulated” (40 and 45 genes, respectively). These results revealed that differentially expressed isoforms with different TSSs offer an additional perspective of gene regulation in LPS-stimulated BV-2 microglial cells.

**Table 2:** Transcriptional and post-transcriptional classification of genes in 2 h LPS-stimulated BV-2 microglial cells.

Un-spliced and transcriptionally regulated genes	Spliced and post-transcriptionally regulated genes	Spliced and both transcriptionally and post-transcriptionally regulated genes
Il1b, Saa3, Cxcl10, Lcn2, Isg15, Ccl4, Ifit1, Fas, Ptgs2, Treml4, Clec4e, Rtp4, Adamts4, Serpine1, Ntng2, Cmpk2, Ccl7, Socs3, Gpr84, Tnfaip2, Slfn2, Parp14, Marco, Prdm1, Mir146, Tnip3, Slc7a11, Ccl3, Dnmt3l, Relb, Zfp703, Bcl2a1d, Cebpd, Bcl2a1b, H2-Q7, Mir221, Rasa4, Gdf15, Tnfsf9, Src, Ccl12, Marcksl1, 2310010J17Rik, Il18, Hp, Cfb, 4933416M07Rik, Hivep2, 1700066J24Rik, Ebi3, Zfp36, Aif1, Pstpip2, Atf3, Fam219a, Samd9l, Ier3, Bcar1, Arhgef3, Rnd1, Kdm4a, Cdkn1a, Birc3, Map3k8, Herpud1, Oasl2, Rab20, Slpi, Plscr1, Ets2, Fkbpl, Rapgef2, Usp18, Dusp2, Tfec, Tnfrsf17, Psd, Trim21, Rassf4, Ngly1, Cd274, Niacr1, Sult6b1, Irak3, Btg2, Mfsd7a, Hivep1, Mir17, Filip1l, Chac2, Cd83, Tmem132a, Irf7, Gpr85, 1700092M07Rik, Rnf19b, Cd14, Ccl9, Rnf19a, Rhoq, Csrnp1, Tm4sf5, Dusp16, Dennd2a, Pou2f2, Ppfibp2, Iqsec2, Nupr1, Stk40, Wnt6, Gemin2, Pion, 8430408G22Rik, 4930487H11Rik, Ppp1r15a, Gimap6, Daam1, Htra1, Ncoa7, H2-Q5, Neurl3, Abtb2, Slc15a3, Gch1, Sowahc, Tmem2, Dcbld2, Sqstm1, Jund, Swap70, Rhob, Rhof, Pik3r6, Tifa, Ccrn4l, Grap, Junb, Eid3, Chst7, Amn1, Ttc32, Lcp2, N4bp1, Rtn2, Nfe2l2, Dtx3l, 2610019E17Rik, Dap, 3110056K07Rik, Zswim4, Vps37c, 4933432I03Rik, Igtp, Bcl10, Agmo, Slc25a25, Rab13, Id3, Plekhh3, 3110043O21Rik, Magohb, Agpat4, Ccdc71l	Irg1, Nos2, Nod2, Tnf, Tnfaip3, Cd40, Ccl2, Rsad2, Pilrb1, Nfkbia, Icosl, Stx11, Sdc4, Oasl1, Ralgds, Clec2d, Irf1, Tnip1, Pilrb2, Lacc1, Rab11fip1, St3gal1, Ehd1, Slc2a6, Lrrc25, Plk2, Maff, Fam49a, Helz2, C3, Rhbdf2, Csf1, Peli1, Itga5, Klf7, Pilra, Tnfrsf1b, Rel, Atp8b4, Xylt2, Fgd3, Nfkbie, Ptafr, Mtmr14, Micall2, Slc11a2, Lilrb4, Trem1, Rhoc, Sod2, Tbc1d2b, 4933426M11Rik, Zfp263, Ksr1, Rasgef1b	Il1a, Traf1, Pde4b, Ccrl2, Nfkbiz, Pdgfb, Pim1, Tlr2, Casp4, Spata13, Trim13, Nlrp3, Nfkb2, Il1rn, Pik3r5, Tank, Adora2b, Cybb, Irak2, Slc31a2, Igsf6, Tet2, Tbce, Snx20, Irf9, C5ar1, Irgm1, Tagap, Ptprj, Mapkapk2, Fam20c, Ppap2a, Osgin2, Malt1, Parp9, Gm2382, Abcc5, A430093F15Rik, Hivep3, Pdlim7

**Table 3:** Transcriptional and post-transcriptional classification of genes in 4 h LPS-stimulated BV-2 microglial cells.

Un-spliced and transcriptionally regulated genes	Spliced and post-transcriptionally regulated genes	Spliced and both transcriptionally and post-transcriptionally regulated genes
Saa3, Cxcl10, Cmpk2, Il1a, Traf1, Isg15, Rtp4, Ifit1, Ccl4, Ptgs2, Cdc42ep2, Mnda, Ccl12, Ccl7, Tnf, Ntng2, Serpina3f, Usp18, Parp14, Clec4e, Hp, Stx11, Ccl2, Tarm1, Slc7a11, Gadd45a, Gpr84, Samd9l, Dhx58, Bcl2a1b, Socs3, Ehd1, Src, Bcl2a1d, Tnip3, Pstpip2, Ebi3, Trim30a, Mir17, Sdc4, Oas3, Pim1, Ifit2, Marcksl1, Tlr3, Slpi, Slfn4, Tnfaip2, Ier3, Oas2, Wnt6, Slfn8, Cebpd, Cd274, Ppfibp2, Icam1, Tnip1, Irgm2, Slc15a3, Adamts4, Ifi203, Relb, Icosl, Gm7609, C3, Plk2, Dnmt3l, Irf1, Gch1, Stk40, Marcks, Fam46c, Mfap3l, Ifi204, Adora2b, Lpcat2, Mndal, Il18, Cd14, Trim12a, 2310034G01Rik, Stat2, Snord11, Serpine1, Lgals9, Itga5, Zfp703, Rab20, Phf11d, Phf11b, Snora41, Ets2, Dcbld2, Dtx3l, Gpr31b, Rhoq, Tor3a, Irak3, Xylt2, Niacr1, Fcgr2b, Herc6, Pion, Trim56, 1810033B17Rik, Tmem132a, Chst7, Ppp1r15a, Gdf15, Cdkn1a, Atf3, Vasp, Notch1, Gm6548, Trim12c, Oas1g, Neurl3, Micall2, A430093F15Rik, Rhoc, Tap1, Kremen1, Apobec3, Rassf4, 4931440P22Rik, Pigv, Procr, Dusp5, Eif2ak2 Csrnp1, Btg2, Tet2, Dusp4, Plaur, Stat1, Fam173b, Rab11fip1, St3gal3, 2010016I18Rik, Nupr1, Daam1, Cdkn2b, Epsti1, Chpf, 9330175E14Rik, Dgat2, Ripk2, Slc25a47, Pcgf5, Trem1, Osgin2, Trafd1, Nfkbie, Lrrc8d, Pex11a, Spata25, Slfn10-ps, Ccl9, Lcp2, Car13, Tagap, Malt1, Junb, Dram1, Rtn2, St3gal1, Scarna8, Kdm4a, AV051173, Rel, Abtb2, Ifi35, Trim14, 4930599N23Rik, Tfec, BC051226, Daxx, Mir677, Rras2, Cebpb, Sbsn, Sqstm1, Arhgef3, Wdr92, Klf7, Filip1l, Dusp2, Gbgt1, Ksr1, Fam20c, Hint2, Tmem2, Phf15, Acsl1, Hck, Cd38, Plekhn1, Rusc2, 4833417C18Rik, Parp12, Map3k8, Lck, Bst2, Hmox1, Slc25a25, Ticam1, Mdm2, Adamts1, Pnp, Zfp36, Osbpl3, Nfe2l2, Rnf19b, Wdr59, Fcgr4, Ppm1k	Irg1, Ccrl2, Oasl2, Pilrb2, Cd40, Nod2, Cfb, Irf7, Tnfaip3, Slfn2, Il1rn, Zc3h12a, Irgm1, Helz2, Pilra, Chpf2, Nfkbiz, Ralgds, Dusp16, Fam49a, Nfkbia, Ikbke, Igsf6, Aoah, 1110038B12Rik, Clec12a, Tnfrsf1b, Slc2a6, Lacc1, Gp49a, Pik3r5, Irak2, Slc31a2, Pik3r6, Mocs1, Sod2, Atrip, Osm, Pou2f2, Parp10, Slc11a2, Ms4a6c, Uba7, 2810474O19Rik, Peli1, Bcl2l11, Tank, Cpd, Rasgef1b, Mtmr14, P2ry2, Snx20, Clec4d, Psmd10, Grap, Slfn3, 4933426M11Rik, Znrf1, Nfkb1, Abcc5, Stard3, Usb1, Ogfr	Rsad2, Pde4b, Pilrb1, Clec2d, Mx2, Oasl1, Pdgfb, Ccl3, Isg20, Casp4, Oas1b, Trim13, Rapgef2, Zc3h12c, Tlr2, Rasa4, Lrrc25, Zufsp, Spata13, Igtp, Nfkb2, Parp9, Zfp719, AI607873, Nlrp3, Znfx1, Birc3, Stap1, Cybb, Oas1a, Ppap2a, Rhbdf2, Rabggtb, Lilrb4, Maff, Irf9, C5ar1, Mapkapk2, Xaf1, Ptprj, Csf1, Adar, Cpeb4, Txnrd1, Ncf4

### Epigenetic regulators in multiple families are novel targets of RNA-Seq data in LPS-stimulated BV-2 microglial cells

**Fig 4:**
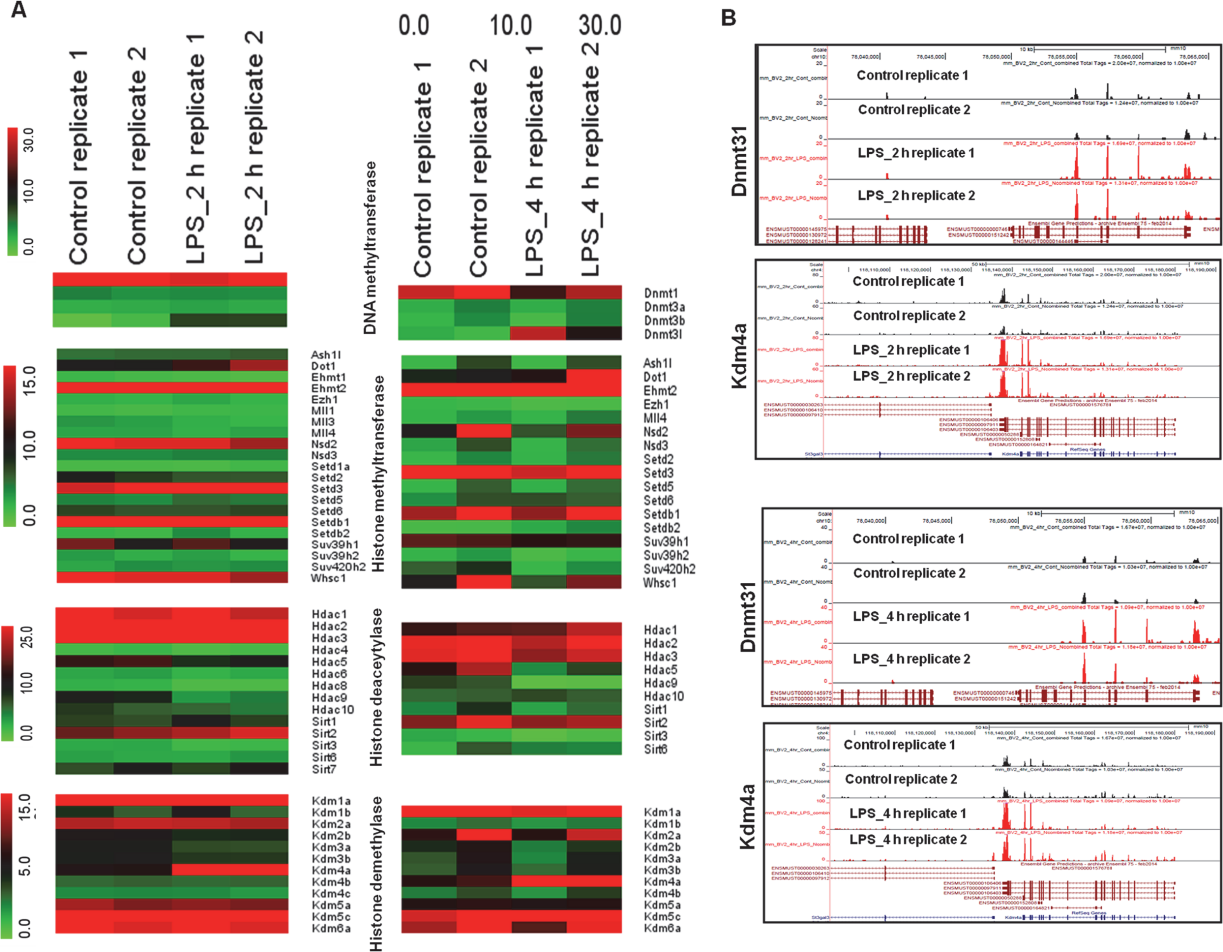
Epigenetic mechanisms for LPS-stimulated BV-2 microglial cells. (A) RNA-Seq analysis of DEGs encoding key enzymes in epigenetic regulation. The heat maps display family-wide gene collections encoding DNA/histone methyltransferases, histone deacetylases and histone demethylases. (B) UCSC Browser images representing normalized RNA-Seq read densities for DNA methyltransferases and histone demethylases at 2 and 4 h after LPS stimulation in BV-2 microglial cells compared with controls.

In addition to differentially expressed TFs, the annotation of the RNA-Seq data also revealed family-wide DEGs implicated in epigenetic regulation, defined as genetic control through factors other than an the DNA sequence [43]. Studies of epigenetic regulation to potentiate innate immune responses have recently emerged [44]. Herein, we provide the first evidence that among multiple families of epigenetic regulators, only histone demethylases (kdm4a) and DNA methyltransferase (dnmt3l) were significantly and differentially expressed in LPS-stimulated BV-2 cells (Figs. 4A and 4B), suggesting that histone demethylases (Kdm4a) and DNA methyltransferases (dnmt3l) might be involved in the regulation of BV-2 microglial cell activation.

### Gene network analysis and canonical pathways modulated through LPS-stimulated BV-2 microglial cells

**Fig 5:**
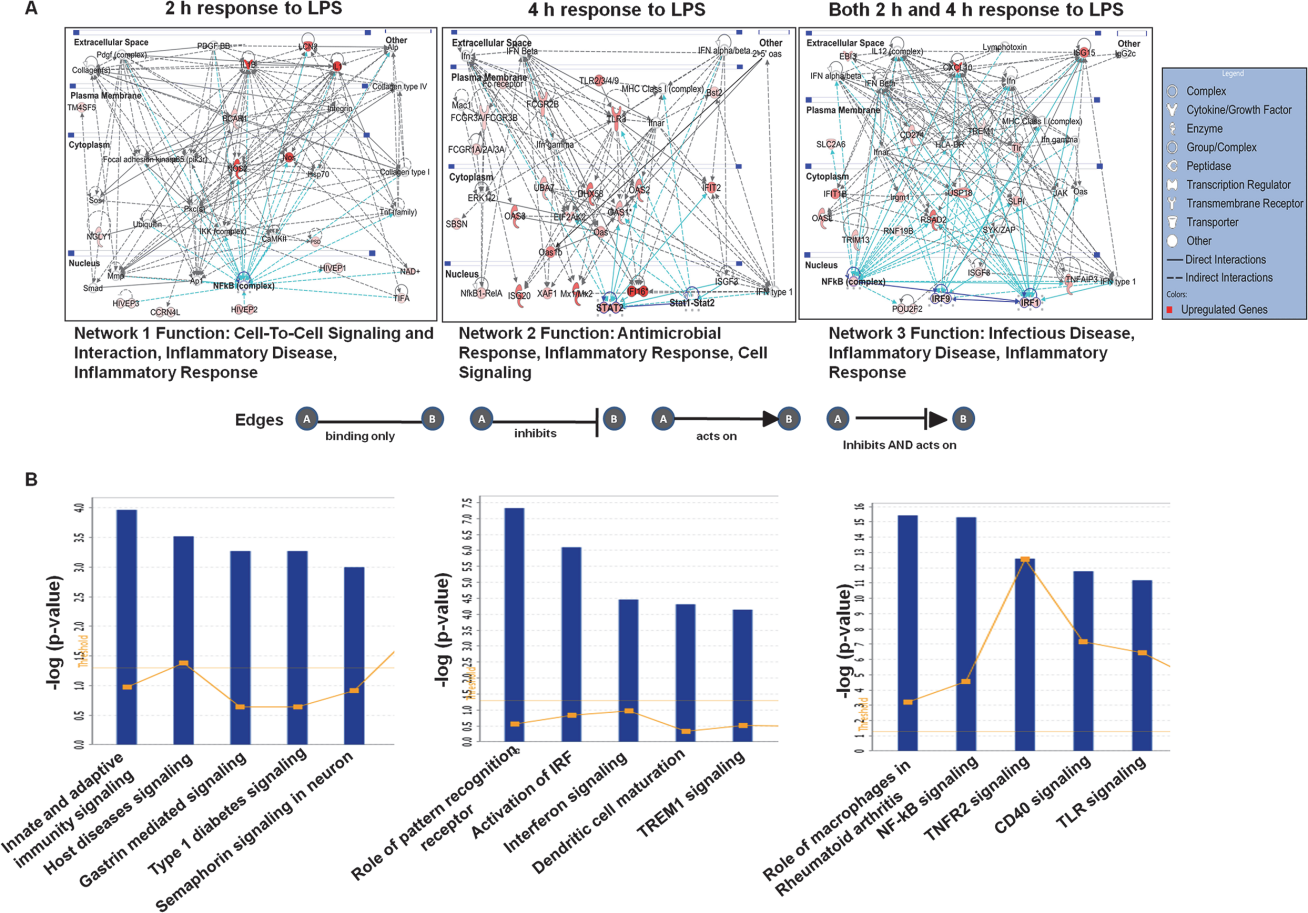
Top network and canonical pathway analyses at each time point (2 h and 4 h) in LPS-stimulated BV-2 microglial cells. (A) Ingenuity Bioinformatics pathway analysis of gene network with connections to nf-κb, stat1-stat2, irf1 and irf9 and differentially expressed genes in LPS-stimulated BV-2 microglia cells. (B) The most highly represented canonical pathways for differentially expressed genes in BV-2 microglial cells.

To gain further insight into molecular functions, we performed IPA to identify gene networks, which represents the intermolecular connections among interacting genes based on functional knowledge inputs. These pathways potentially define molecular targets associated with LPS-stimulated BV-2 cells. The IPA analysis revealed three networks of the differentially expressed genes established either at 2 or 4 h or at both time points after LPS stimulation in BV-2 cells. Network 1, network 2 and network 3 are illustrated in Fig. 5A. The genes in networks 1, 2 and 3 were involved in cell-to-cell signaling and interaction, inflammatory disease, and inflammatory response at 2 h after LPS stimulation in BV-2 cells; antimicrobial response, inflammatory response, and cell signaling at 4 h after LPS stimulation in BV-2 cells; and infectious disease, inflammatory disease, inflammatory response at both 2 and 4 h after LPS stimulation in BV-2 cells. Interestingly, the genes enriched in LPS-stimulated BV-2 cells were in networks, and nf-κb complex, stat1-stat2, irf1 and irf9 formed the central node of an interconnected regulatory system. Among these genes, nf-κb target genes, such as proinflammatory cytokines, Il1ß, nos2; stat1-stat2 target genes, such as oas1, oas2, and ifit2; and irf1 and irf9 target genes, such as cxcl10, isg15, rsad2, and slpi, have been implicated in inflammatory disorders (Fig. 5A). The IPA-identified biological networks and pathways most involved in LPS-stimulated BV-2 cells complemented the findings from Pathway Express. We next examined the modulated genes in canonical pathways by mapping these transcripts to the IPA program. The top 5 canonical pathways for the differentially expressed genes at the two time-points are displayed in Fig. 5B. The most significant canonical pathways were involved in communication between innate and adaptive immune cells and pattern recognition receptors in recognition bacteria and viruses. Other notable pathways included the activation of Irf through cytokine pattern recognition receptors and interferon, NF-κB, Tnfr2 and CD40 signaling in LPS-stimulated BV-2 cells. Furthermore, the diseases, disorders and upstream regulators for LPS-stimulated BV-2 cells are shown in Tables 4 and 5.

**Table 4:** Top diseases and disorders identified through Ingenuity Pathway Analyses.

	Top diseases and disorders	*p-*value	No. of molecules
**A**	Inflammatory response	2.93E-05-7.72E-03	22
	Gastrointestinal diseases	4.40E-05-7.72E-03	9
	Hepatic system diseases	4.40E-05-7.72E-03	2
	Cancer	2.18E-04-7.72E-03	47
	Hematological diseases	2.18E-04-7.72E-03	12
**B**	Immunological diseases	7.42E-15-5.92E-03	42
	Antimicrobial response	3.25E-14-2.91E-04	19
	Inflammatory response	3.25E-14-6.46E-03	48
	Infectious diseases	1.67E-12-6.46E-03	45
	Endocrine system disorders	2.89E-12-4.62E-08	25
**C**	Infectious diseases	7.63E-29-1.65E-06	80
	Inflammatory response	4.01E-28-1.81E-06	90
	Hematological diseases	9.10E-18-1.34E-07	50
	Connective tissue disorders	3.34E-17-8.68E-07	50
	Inflammatory diseases	3.34E-17-1.45E-06	73

A) 2 h response to LPS; B) 4 h response to LPS; C) response to LPS at both 2 and 4 h in BV-2 microglial cells.

**Table 5:** Top upstream regulators identified through Ingenuity Pathway Analyses.

	Top upstream regulators	*p-*value	Predicted activations
**A**	Lipopolysaccharide	9.11E-13	Activated
	Tlr4	4.52E-08	Not Activated
	Ifn	6.67E-08	Activated
	Resiquimod	8.66E-08	Not Activated
	Irf8	1.39E-07	Not Activated
**B**	Lipopolysaccharide	3.34E-28	Activated
	Poly I.C RNA	2.98E-26	Activated
	Irf7	1.35E-22	Activated
	Ifnb1	9.21E-22	Activated
	Ifna2	5.14E-20	Activated
**C**	Lipopolysaccharide	9.43E-71	Activated
	Ticam1	3.29E-59	Activated
	Il1b	6.80E-59	Activated
	Salmonella minnesota R595 lipopolysaccharide	1.10E-54	Activated
	Ifng	2.53E-53	Activated

A) 2 h response to LPS; B) 4 h response to LPS; C) response to LPS at both 2 and 4 h in BV-2 microglial cells.

### Confirmation of differentially expressed genes through qRT-PCR

**Fig 6:**
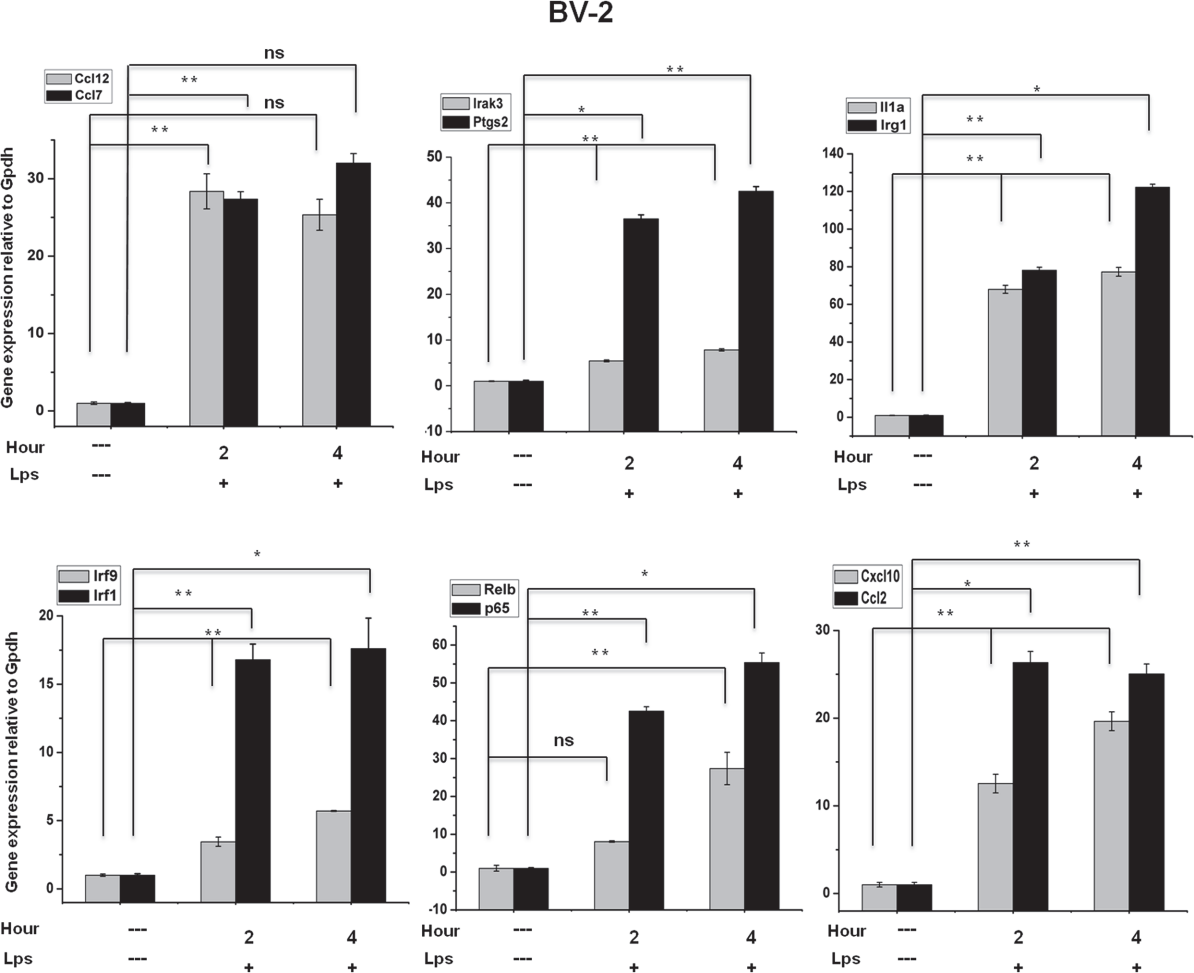
Confirmation of differentially expressed genes by quantitative reverse transcription-polymerase chain reaction in BV-2 microglial cells. ccl12, ccl7, irak3, ptgs2, il1a, irg1, irf9, irf1, relb, p65, cxcl10, and ccl2 genes were significantly up-regulated in LPS-treated BV-2 microglia cells. Gene expression was normalized to the GAPDH transcript levels. *P<0.*05*, **P<0.*001* and ns stands for no significant difference compared with control. The data represent three independent experiments.

**Fig 7:**
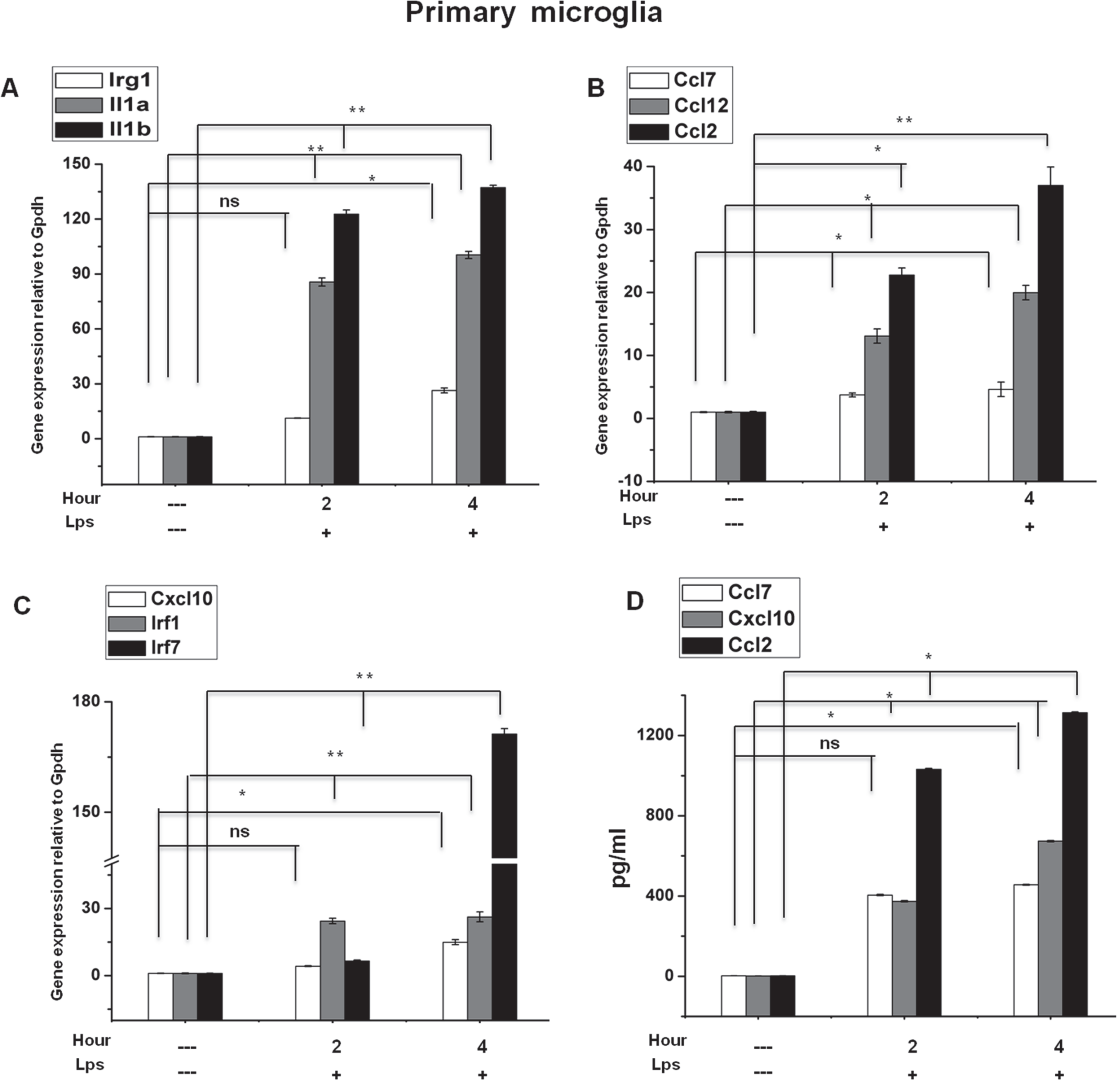
Confirmation of differentially expressed genes and release of pro-inflammatory mediators in primary microglial cells. (A, B, and C) irg1, il1a, il1b, ccl7, ccl12, ccl2, cxcl10, irf1, and irf7 genes were significantly up-regulated in LPS (10 ng/mL) treated primary microglia cells. Gene expression was normalized to the GAPDH transcript levels. (D) Primary microglial cell culture supernatants of LPS (10 ng/mL) treated cells were subjected to ELISA to detect the levels of pro-inflammatory cytokines/chemokines. Therefore, primary microglial cells were treated with LPS for 2 h and 4 h, followed by quantification of ccl2, ccl7, and cxcl10 levels. Values are given in pg/ml. Means and standard deviations of the mean of three independent experiments are shown (*P value <0.*05*, **P value <0.*001*, ns stands for no significant difference compared with control).

A large number of differentially regulated genes identified in the RNA-Seq analysis were subjected to validation through real-time qRT-PCR using GAPDH as a reference gene. The LPS-affected genes were primarily selected for validation. To measure gene expression, mRNA was reverse transcribed into cDNA using the Prime Script TM Reverse Transcriptase (Takara Bio Inc., Shiga, Japan); the qRT-PCR assays were repeated several times using at least 3 mRNA preparations from independent experiments. The results are expressed as a fold-change relative to the control levels. Fifteen genes were selected for verification; the RNA-Seq expression patterns were confirmed for twelve genes (ccl12, ccl7, irak3, ptgs2, il1a, irg1, irf9, irf1, relb, p65, cxcl10, and ccl2; Fig. 6), and three genes were non-significant (data not shown) in the qRT-PCR analysis compared with the RNA-Seq experiments. To confirm whether those genes were induced in primary microglial cells, we incubated primary microglial cells under inflammatory conditions (LPS 10 ng/mL), which induced inflammatory genes including irg1, il1a, il1b, ccl7, ccl12, ccl2, cxcl10, irf1, and irf7 in primary microglial cells (Figs. 7A, 7B and 7C). However, it should be noted that ptgs2 gene was not affected by the treatment of LPS (data not shown). In addition, we analyzed cytokines/chemokines in the supernatants of treated primary microglial cells with ELISAs. Compared to untreated cells ccl2, ccl7, and cxcl10 in the supernatants were increased in primary microglial cells following 2 h and 4 h LPS (10 ng/mL) treatment (Fig. 7D).

### Differential impact of Aβ_42_ on the expressions of inflammatory mediators in microglial cells

**Fig 8:**
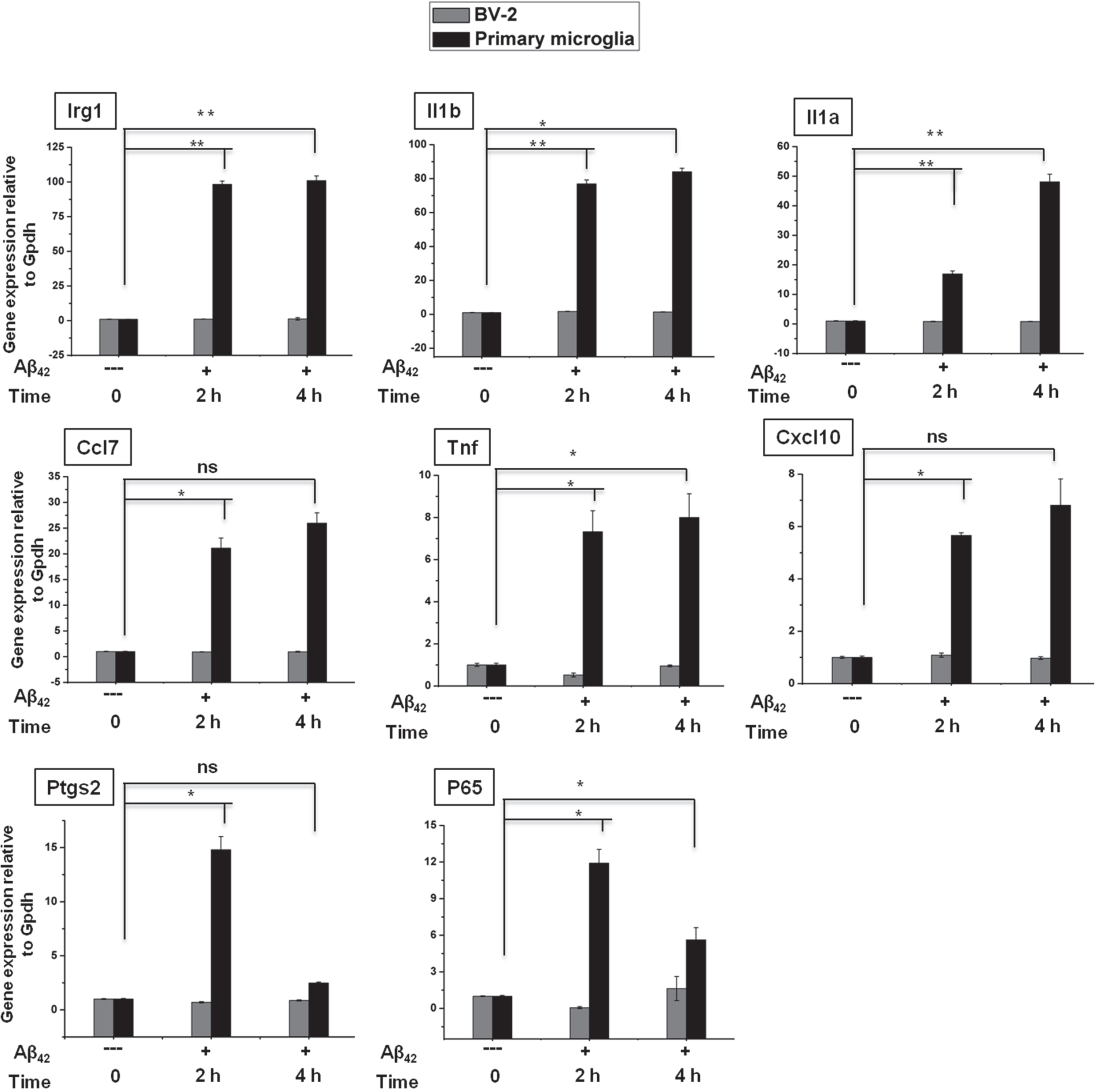
Effect of Aβ_42_ on the expressions of inflammatory mediators in microglial cells. Quantitative real-time reverse transcriptase-PCR analysis of inflammatory gene expression in BV-2 and primary microglial cells stimulated with Aβ_42_. The expression of inflammatory genes were significantly up-regulated in primary microglial cells treated with Aβ_42_ compared with untreated cells (*P<0.*05*, **P<0.*001* and ns stands for no significant difference compared with control) at the indicated times. Gene expression was normalized to GAPDH transcript levels. The data represent three independent experiments. The values are shown as the means ± SD of triplicate wells.

Previous studies have demonstrated that Aβ_42_ is a principal component of senile plaques and is thought to be central to the pathogenesis of the AD [45, 46] and abundant proinflammatory cytokines, chemokines, and complement products are presented in AD brains [47, 48]. To probe whether Aβ_42_ triggered deregulation of inflammatory genes, we measured the expressions of selected inflammatory genes upon exposure to Aβ_42_ for 2 h and 4 h time points in both BV-2 microglial and primary microglial cells. Interestingly, we found that most of the inflammatory response-related genes were significantly up-regulated only in primary microglial cells at the 2 and 4 h time points upon exposure to Aβ_42_ (Fig. 8). However, it should be noted that irf1, irf7, ccl2, ccl12, and relb genes were not affected by the treatment of Aβ_42_ (data not shown). Nevertheless, in the presence of chronic Aβ_42_ exposures detailed transcriptome analysis will be required to determine the unique transcriptomic signature in primary microglial and BV-2 microglial cells. This is an exciting area that we are keenly pursuing further.

## Discussion

In the present study, we used an RNA-Seq approach to profile the differential gene expression at multiple time-points in BV-2 microglial cells in response to inflammatory stimulus. Although other methods, such as microarray technology, have been applied for the genome-wide analysis of inflammatory gene transcription in macrophages, the experimental strategy described here provided novel insights into high-resolution transcriptome data [20, 30, 45]. RNA-Seq technology, combined with bioinformatics, is an efficient high-throughput tool to establish gene expression patterns and complete functional clustering, canonical pathway and network enrichment of DEGs, with great advantages for identifying host response genes and obtaining associated information following LPS infection. Bioinformatics analyses of DEGs revealed approximately 263 and 319 genes (≥ 1.5 log_2_-fold) were significantly up-regulated after 2 and 4 h, respectively, in LPS-stimulated BV-2 cells. This variation between 2 h and 4 h time point may be due to relatively less immune response gene expression level at its earliest stage of bacterial infection when high variation is more pronounced compared to the 4 h time point. Notably, these data were accurate, although two independent biological replicates for each sample were used. In addition, we investigated the use of differential promoters, transcription start sites and isoforms variants in LPS-stimulated gene loci, which, to our knowledge, has not been previously validated in studies concerning genome-wide gene regulation in microglia [49, 50].

The analysis revealed that the genes associated with inflammation were significantly up regulated in response to LPS in BV-2 microglial cells. Both the extent of the fold-change and the number of genes were significantly modulated. The following inflammatory response- and immune response-related genes were markedly affected after LPS stimulation: iNOS, interleukin and interleukin-related genes (il1-β, il1a, il18, and il1rn); Tnf and Tnf-related genes (tnf-α, tnfaip3, tnip3, tnip1, and tnfaip2); a prostaglandin-related gene, ptgs2; nf-κb-related genes (nfκbiz, nfκbia, nfκb2, relb, nfκbie, and nfκb1); interferon-related genes (ifit1, irf1, irf7, irf9); and cytokines or chemokines (cxcl10, ccl4, ccl7, ccl2, ccl3, ccl12, and ccl9) (Fig. 2A).

We observed that LPS significantly induced the expression of key pro-inflammatory enzymes, including nos2 and ptgs2. Nos2 plays a pivotal role in mediating neuroinflammation to produce NO, a potent proinflammatory mediator, via oxidative deamination [51]. Because neurons and oligodendrocytes are injurious in relation to NO, an oversupply of NO can cause nerve injury in CNS diseases [52]. Ptgs2 is the key enzyme responsible for brain inflammation, and increased ptgs2 expression contributes to neurodegeneration [53]. In addition, Ptgs2 is also responsible for the synthesis of inflammation-related PG, and the inhibition of PG and NO production has been proposed as a therapeutic target for inflammatory diseases, such as PD, Huntington’s disease and AD [54]. Chemokines, also referred to as inflammatory cytokines, are key regulators of inflammation, and the excessive production of these molecules has been associated with disease progression and severe inflammation pathologies, including MS [55]. Conductier *et al*. reported that ccl2 plays a crucial role in neuroinflammatory diseases and is also considered as a target in the treatment of neuroinflammatory disorders [56]. Ccl2 and ccl7 are highly expressed during MS in microglia, astrocytes and other inflammatory cells [57]. Ccl12 also plays an inflammatory role, as the levels of this chemokine are up-regulated in both microglia and astrocytes when stimulated with the proinflammatory cytokine il-17 [58]. The expression of the CXC chemokine ligand 10, cxcl10, is observed during infectious and inflammatory diseases, playing a crucial role in T-cell mediated inflammation in the CNS. In addition, Cxcl10 plays a role in inflammatory demyelinating diseases, such as MS, through the destruction of the myelin sheath or neurons by facilitating leukocyte trafficking in the brain [59]. A previous study reported that rabies virus infection up-modulated the expression of interferon-stimulated genes (ISGs), such as ifit1, ifit2, ifit4, isg20, gbp5, gbp1, oas1, oas3 and mxa, in NT2-N cells [60]. In the present study, we established the profound up-regulation of some ISGs, such as oasl1, oasl2, irf1, irf7, isg15 and igtp, in microglial cells at 2 and 4 h after LPS stimulation. This result suggested that LPS infection caused the activation of IFN-signaling-pathway-induced gene expression in BV-2 microglial cells, although the modulation of IFN-α/ß genes was not detected in the RNA-Seq analysis. Furthermore, to evaluate the influence of microglial cells on Aβ_42_-induced AD [45–48] we measured the expressions of selected inflammatory genes upon exposure to Aβ_42_ for 2 h and 4 h time points in both BV-2 microglial and primary microglial cells. Interestingly, we found that most of the inflammatory response-related genes were significantly up-regulated in primary microglial cells at the 2 and 4 h time points (Fig. 8). However, BV-2 cell lines with these factors did not induce the expression of such inflammatory response-related genes.

Another hallmark of inflammation is the increased expression of TFs. The RNA-Seq analysis identified several groups of TFs most relevant for microglia activation. Roles have been established for most of these TFs, including nf-kb (global activator), stat, spi1 (Pu.1), and Irfs, in macrophage activation [61]. In addition to nf-κb and stat we identified additional TFs (spi1 (Pu.1), irfs, klf7, junb, atf3, and foxp4) whose roles in microglia activation have not yet been established (Fig. 3A, 3B). We observed the significant up-regulation of stat1 and stat3 after 4 h in LPS-stimulated BV-2 microglia. Similar observations were also observed in peripheral macrophages stimulated with LPS, revealing the strong up regulation of stat1/stat3 signaling [62]. The RNA-Seq data also revealed that other members of these transcription factor families, including klf7, junb, atf3, and foxp4, could play significant roles in microglia development and activation. However, irf2, irf4, irf6, irf8, stat6, klf1, klf2, klf4, and klf5 were unaffected by LPS, suggesting the highly selective induction of TFs through LPS in BV-2 microglial cells. Nevertheless further studies are warranted to assess earlier timepoint transcription factors profiling as well as how these TFs participate in innate immunity in microglial cells. Furthermore, we confirmed the expression of key inflammation- and immunity-related genes as well as cytokines/chemokines in the supernatants were significantly induced in LPS treated primary microglial cells including irg1, il1a, il1b, ccl7, ccl12, ccl2, cxcl10, irf1, and irf7 (Figs. 7A, 7B, 7C and 7D).

To further delineate conserved transcription factor-binding motifs, we performed TF motif analysis on LPS-stimulated genes in BV-2 microglial cells. The core promoters of co-expressed genes (typically, regulatory regions within −1000 to +50 bp relative to the transcriptional start site) can be evaluated for overrepresented cis-regulatory elements after partitioning into suitable modules [63]. Among the two ranges available in Pscan that are closest to this region of interest (−950 to 50 and −1000 to 0), the −950 to +50 bp range was selected for the analyses. The promoters of differentially expressed genes revealed the enrichment of DNA sequences not only for NF-κB transcription factors but also for irf1, stat1, stat3 and specificity protein 1 (Spi1). These analyses converged the first insights into 5 TF binding motif that can be involved in regulating subset specific genes in BV-2 microglial cells (Fig. 3D).

To further elucidate the functional categories for LPS-stimulated inflammatory genes in BV-2 microglial cells, we performed the first functional analysis of the transcripts, isoforms and TSSs. Most of the differentially expressed genes in microglial following LPS stimulation were expressed as several isoforms subjected to transcriptional/post-transcriptional regulation and/or differential promoter usage. We classified these genes into three main groups (genes with one isoform and one TSS; genes with more than one isoform and one TSS; and genes with more than one isoform and more than one TSS (Fig. 3E and Tables 2 and 3). The first two groups included genes crucial for the innate immune response, which might be under stronger selection to prevent the emergence of new isoforms and/or post-transcriptional regulation. In addition few of the genes [64] belonged to the third group, suggesting that they could be subjected to positive selection at the transcriptional and post-transcriptional regulation in LPS stimulated BV-2 microglial cells. However, further targeted studies are required to validate this regulation and establish the potential effects of these genes during microglial infection.

Epigenetic regulation, which involves chemical modification of DNA cytosine residues and DNA-bound histone proteins without alterations in the DNA sequence, is promising as one of the major factors regulating gene expression in response to environmental stimuli [43]. Recent studies have demonstrated that histone demethylases (kdm6b) and histone deacetylases (hdac1, hdac2, hdac3, and hdac7) potentially regulate proinflammatory gene expression in macrophages [44, 65, 66]. Recently, we showed that the histone demethylase kdm4a was significantly expressed in neuroectodermal stem cells and might play a role in tumorigenic development [35]. Interestingly herein, the RNA-Seq data also revealed that the histone demethylase Kdm4a and DNA methyltransferase Dnmt3l were strikingly differentially expressed in LPS-stimulated BV-2 microglial cells (Figs. 4A and 4B). However, the histone demethylase Kdm6b and histone deacetylases, hdac1, hdac2, hdac3, and hdac7, were not affected in LPS-stimulated BV-2 microglial cells.

The top KEGG pathways identified in DAVID included immune system processes and stimuli responses, while the top canonical pathways identified in IPA involved the communication between innate and adaptive immune cells and pattern recognition receptors in recognition bacteria and viruses. Furthermore, the most pronounced functional network over-represented in these data involved NF-κB complex, stat1-stat2, irf1 and irf9, which form the central molecule of an interconnected regulatory system, suggesting that NF-κB complex, stat1-stat2, irf1 and irf9 link proinflammatory cytokines, il1ß, nos2, oas1, oas2, ifit2, cxcl10, isg15, rsad2, and slpi to mediate the signaling and induction of proinflammatory activities in microglia in response to LPS stimulation (Fig. 5A). The up-regulation of NF-κB complex, stat1-stat2, irf1 and irf9 in LPS-stimulated BV-2 microglial cells was further illustrated in the differential gene expression analysis (Figs. 2A and 2B). In the present study, we examined BV-2 cell lines as a model of inflammation studies. This is one of the major uses of microglia. Previously, others reports demonstrated that BV-2 cell lines have close resemblance to primary brain microglia [67–69]. Consistent with our findings, Henn *et al*. reported that in the presence of LPS transcriptome and proteome analysis of BV-2 cell lines revealed a high similarity to primary microglial cells [69]. Since BV-2 cells are easy to culture, they are an important tool to study not only inflammatory processes, [69] but also phagocytosis [70]. Recently, Crotti *et al*. reported that BV-2 cell lines exhibit many similarities to that of primary microglia and in vivo in terms of Huntington's disease [71]. In contrast, Butovsky *et al*. demonstrated that in different conditions, such as after exposure to macrophage colony-stimulating factor (MCSF) and transforming growth factor beta 1 (TGF-ß1) adult primary microglia showed a unique molecular expression pattern. However, MCSF and TGF-ß1 did not induce such microglial molecular expression pattern in BV-2 cell lines [72]. In the presence of LPS as well as MCSF and TGF-ß1 detailed transcriptome analysis will be required to determine the unique transcriptomic signature in primary microglial cells.

Overall, the genome-wide analysis through RNA-Seq showed LPS-inducible genes in microglial cells, reflecting the robust and reliable kinetic development and modulation of cell reactivity during the early course of the inflammatory response. Regardless of certain boundaries in exactitude, LPS-stimulated inflammatory gene expression profiling in microglia, clustering, and the prediction of cis-regulatory elements offer valuable information for future studies, such as potential gene targets for chromatin immunoprecipitation (ChIP)-seq assays. This model can subsequently be extended to include data from other high-dimensional surveys, such as microRNA, ChIP-on-chip, and proteomics, providing more advanced insight into global gene regulation in BV-2 microglial cells.

## Conclusion

In summary, in addition to identifying potential signature genes using RNA-Seq, our pathway analysis discovered multiple pathways and gene networks significantly involved inflammatory disorders in response to LPS stimulation in BV-2 microglia at different activation statuses. Furthermore, we examined epigenetic regulators and the transcriptional and post-transcriptional regulation of genes based on their isoforms, TSSs and differential promoters in LPS-stimulated BV-2 microglial cells. Consequently, we suggest that the panel of microglial genes, TFs, and epigenetic regulators whose expression is differentially regulated in response to bacterial infection may play a critical role for initiating the inflammation process in BV-2 microglial cells and may also contribute to the inflammation-mediated neurodegenerative diseases.

## Supporting Information

S1 Fig Quantification of CD11b positive microglial cells. Microglial identification is accomplished using flow cytometry. 96.27% of cells obtained were microglia as quantified by CD11b. The labeled cells are represented by the pink shaded populations.
(TIF)

S2 Fig Functional annotations of LPS-inducible down-regulated genes. (A and B) Gene Ontology analysis of functional annotations (biological process) associated with 2 and 4 h LPS-inducible down-regulated genes in BV-2 microglia in comparison with the control, respectively.
(TIF)

S1 File Contains Tables A and B. Top 25 significant down-regulated genes in 2 and 4 h LPS stimulated BV-2 microglial cells.
(DOC)
