# Supplementary figures and images for: Dual RNA Sequencing Reveals the Expression of Unique Transcriptomic Signatures in Lipopolysaccharide-Induced BV-2 Microglial Cells
Source: PLoS One. 2015 Mar 26;10(3):e0121117. doi: 10.1371/journal.pone.0121117 (PMC4374676; doi:10.1371/journal.pone.0121117)

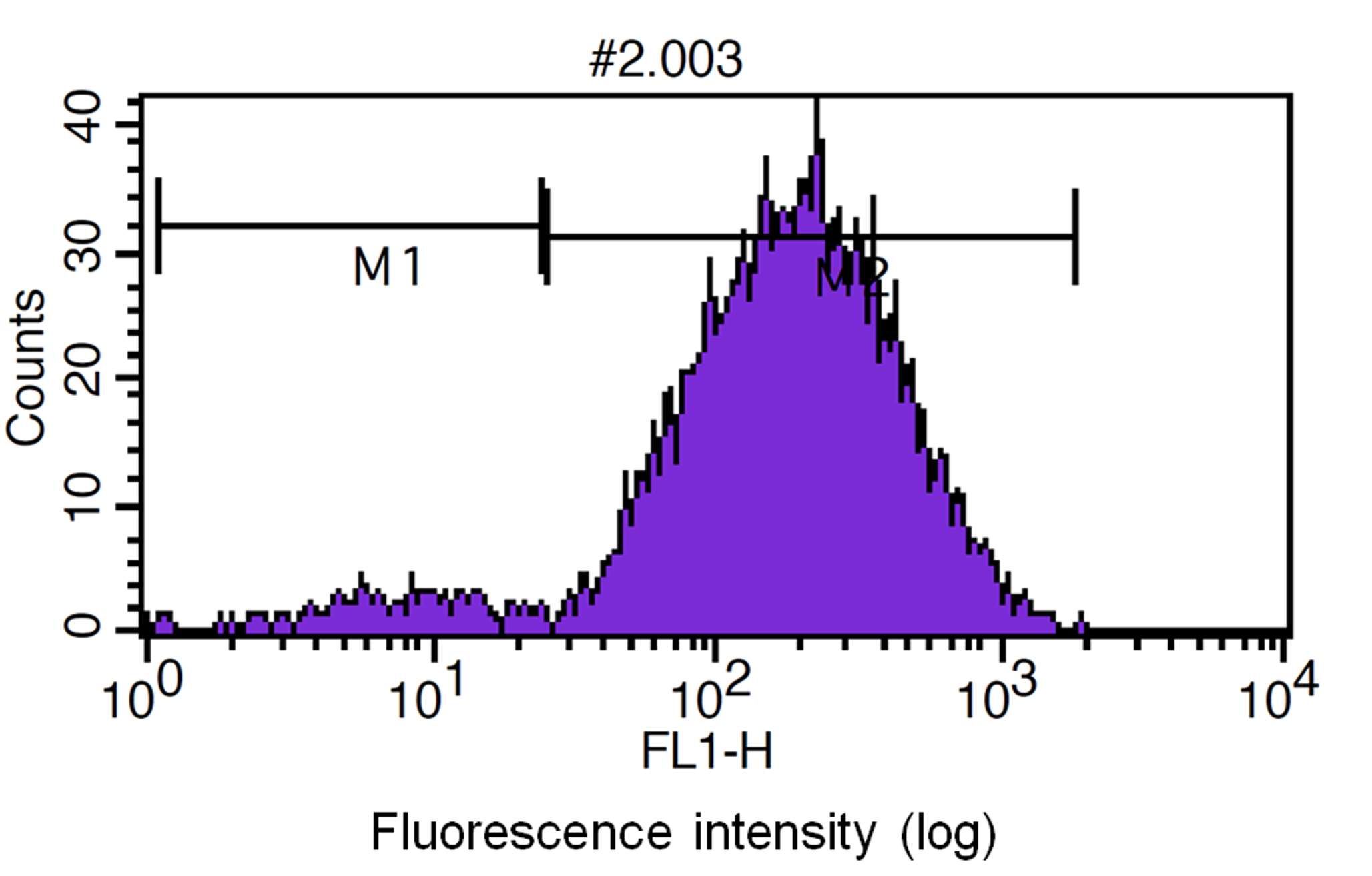

Supplement: S1 Fig — Microglial identification is accomplished using flow cytometry. 96.27% of cells obtained were microglia as quantified by CD11b. The labeled cells are represented by the pink shaded populations. (TIF) [file pone.0121117.s001.tif]

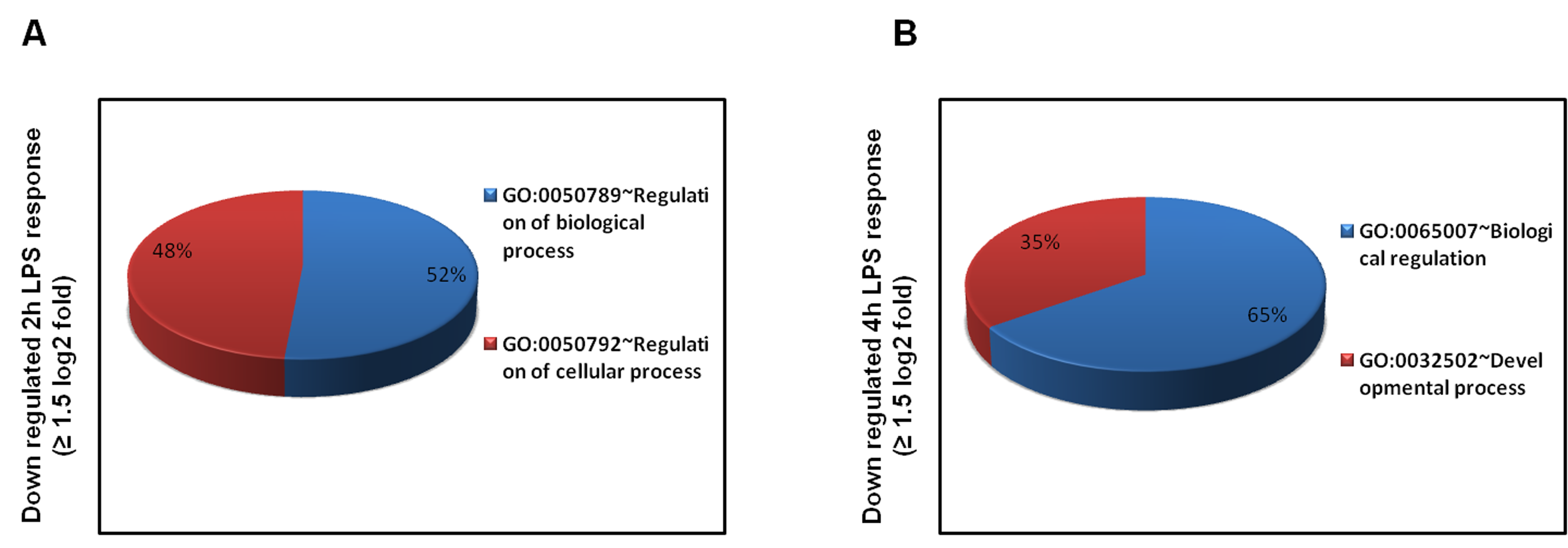

Supplement: S2 Fig — (A and B) Gene Ontology analysis of functional annotations (biological process) associated with 2 and 4 h LPS-inducible down-regulated genes in BV-2 microglia in comparison with the control, respectively. (TIF) [file pone.0121117.s002.tif]
